# Diagnosis of Alzheimer’s disease via resting-state EEG: integration of spectrum, complexity, and synchronization signal features

**DOI:** 10.3389/fnagi.2023.1288295

**Published:** 2023-11-07

**Authors:** Xiaowei Zheng, Bozhi Wang, Hao Liu, Wencan Wu, Jiamin Sun, Wei Fang, Rundong Jiang, Yajie Hu, Cheng Jin, Xin Wei, Steve Shyh-Ching Chen

**Affiliations:** ^1^Expert Workstation in Sichuan Province, Chengdu Jincheng College, Chengdu, China; ^2^School of Mathematics, Northwest University, Xian, China; ^3^Medical Big Data Research Center, Northwest University, Xi'an, China; ^4^School of Humanities and Education, Xi'an Eurasia University, Xi'an, China; ^5^Institute of Social Psychology, Xi'an Jiaotong University, Xi'an, China

**Keywords:** Alzheimer’s disease (AD), electroencephalogram (EEG), spectrum, complexity, synchronization, supervised machine learning

## Abstract

**Background:**

Alzheimer’s disease (AD) is the most common neurogenerative disorder, making up 70% of total dementia cases with a prevalence of more than 55 million people. Electroencephalogram (EEG) has become a suitable, accurate, and highly sensitive biomarker for the identification and diagnosis of AD.

**Methods:**

In this study, a public database of EEG resting state-closed eye recordings containing 36 AD subjects and 29 normal subjects was used. And then, three types of signal features of resting-state EEG, i.e., spectrum, complexity, and synchronization, were performed by applying various signal processing and statistical methods, to obtain a total of 18 features for each signal epoch. Next, the supervised machine learning classification algorithms of decision trees, random forests, and support vector machine (SVM) were compared in categorizing processed EEG signal features of AD and normal cases with leave-one-person-out cross-validation.

**Results:**

The results showed that compared to normal cases, the major change in EEG characteristics in AD cases was an EEG slowing, a reduced complexity, and a decrease in synchrony. The proposed methodology achieved a relatively high classification accuracy of 95.65, 95.86, and 88.54% between AD and normal cases for decision trees, random forests, and SVM, respectively, showing that the integration of spectrum, complexity, and synchronization features for EEG signals can enhance the performance of identifying AD and normal subjects.

**Conclusion:**

This study recommended the integration of EEG features of spectrum, complexity, and synchronization for aiding the diagnosis of AD.

## Introduction

According to the World Health Organization (WHO), more than 55 million individuals currently live with dementia, a number projected to increase to 78 million by 2030 and a staggering 139 million by 2050 (WHO, 2021). Alzheimer’s disease (AD), a neurological disorder, constitutes the predominant form of dementia, accounting for approximately 70% of cases in the world (Blennow et al., 2006). AD mainly occurs in people aged 65 and older, with its incidence rate notably escalating as age advances (McKhann et al., 1984). Due to the high prevalence of AD and its effect on economic cost, WHO has issued a call to prioritize dementia on global health agendas to heighten awareness, enhance early diagnosis, and offer improved care and support to individuals affected by dementia (Subedi and Sapkota, 2019).

Diagnosis of AD, and in particular early diagnosis is essential due to several reasons (Brookmeyer et al., 2007; Dauwels et al., 2010; Galimberti and Scarpini, 2011): (1) it gives patients a warning effect; (2) symptoms-delaying medications are most effective at an early stage of the disease; (3) effective management of psychiatric symptoms, such as depression or psychosis, holds the potential to alleviate the societal burden and associated costs; (4) preventive therapies may be developed to raise the chance of treating the AD. Thus far, diagnosing AD typically involves a comprehensive approach that combines extensive testing and the systematic elimination of alternative potential causes. Psychological assessments, e.g., mini-mental state examinations (MMSE; Folstein et al., 1975) and Montreal cognitive assessment (MoCA; Nasreddine et al., 2005), blood tests (Moretti, 2015), cerebrospinal fluid (CSF; Jack et al., 2011), and emerging imaging techniques are being employed to diagnose AD (Weiner, 2009).

In recent decades, neuroimaging tools, e.g., magnetic resonance imaging (MRI; Dickerson and Wolk, 2011), positron emission tomography (PET; Risacher et al., 2021), and computed tomography (CT; Imabayashi et al., 2013), have been extensively employed to investigate the underlying causes of AD and to enhance the precision of its diagnosis. However, patients receive a diagnosis based on the present spatial resolution of these neuroimaging techniques, often after notable neurodegeneration has occurred. Additionally, these advanced neuroimaging methods come with considerable expenses, demand time-intensive investment, and necessitate experts for their proper intervention.

Electroencephalogram (EEG), an alternative approach that offers greater ease and convenience, has been used as a biomarker in AD diagnosis, due to its low cost, wide availability, high resolution, and high efficiency (Cassani et al., 2018). By measuring the brain’s electrical activity, EEG can detect anomalies in brain waves associated with specific disorders (Noachtar and Rémi, 2009; Kemp et al., 2010; Zheng et al., 2019). Given that EEG signals can reflect functional alterations in the cerebral cortex, EEG-based biomarkers hold the potential to evaluate neuronal degeneration caused by AD progression even before the manifestation of behavioral symptoms (Miltiadous et al., 2021). EEG offers many perspectives from recorded signals, including frequency, dynamic alterations, and source imaging. Previous studies have proven these three typical effects, i.e., diffuse slowing, reduced complexity, and decreased synchronization, of AD patients on resting-state EEG signals compared to normal subjects (Cassani et al., 2018). Firstly, diffuse slowing of brain activity refers to a phenomenon where the power of higher EEG frequency bands (e.g., alpha, beta, and gamma bands) decreases, while the power of lower EEG frequency bands (e.g., delta and theta bands) increases (Jeong, 2004; Garn et al., 2015). Secondly, reduced complexity means the complexity of the brain’s electrical activity decreases in AD patients when compared to healthy individuals (Schätz et al., 2013; Şeker et al., 2021). Thirdly, decreased synchronization manifests as a decline in connectivity between different cortical regions in many AD patients (Koenig et al., 2005; Wen et al., 2015).

After extracting the EEG features by signal processing methods, using the machine learning techniques, e.g., decision trees algorithm, K-nearest neighbors (kNN), regularized linear discriminant analysis (RLDA), and support vector machine (SVM), these features can be automatically analyzed to classify the normal and abnormal (Fiscon et al., 2018; Safi and Safi, 2021). However, the automatic identification of AD through the utilization of machine learning and EEG readings is currently in its early stages and lacks research about the effect on diagnosis performance from the integration of various types of EEG features (Dauwels et al., 2010).

On this basis, this study aimed to explore the EEG characteristics of AD patients and then develop a new diagnostic approach for AD with various types of EEG signal features and supervised machine learning classification methods based on a big public database. First, according to previous studies, the EEG signal features of spectrum, complexity, and synchronization, of AD and normal subjects were obtained. Then, combined with the machine learning algorithms of SVM, decision trees, and random forest, the classification results between AD and normal subjects were acquired by leave-one-person-out cross-validation.

## Methods

### Database description

The public database containing the resting-state EEG recordings from 36 AD patients (aged 66.4 ± 7.9 years, 24 females) and 29 healthy controls (CN; aged 67.9 ± 5.4 years, 11 females) was used in this study (Miltiadous et al., 2023). No other dementia-related comorbidities have been reported in AD patients. The cognitive and neuropsychological assessment was conducted using the MMSE (Creavin et al., 2016). MMSE score ranges from 0 to 30, where a lower score indicates a more severe cognitive decline. The MMSE for the AD group was 17.75 ± 4.5 and for the CN group was 30.

EEG Recordings were collected from 19 scalp electrodes (Fp1, Fp2, F7, F3, Fz, F4, F8, T3, C3, Cz, C4, T4, T5, P3, Pz, P4, T6, O1, and O2) along with 2 reference electrodes (A1 and A2), conforming to the 10–20 international system (Homan et al., 1987). Each recording adhered to the established clinical protocol with participants having their eyes closed. Each recording lasted approximately 13.5 min for the AD group (min = 5.1, max = 21.3), and 13.8 min for the CN group (min = 12.5, max = 16.5). The sampling rate was 500 Hz.

### Signal preprocessing

Firstly, the signals were re-referenced to A1-A2. Secondly, the Butterworth band-pass filter within the frequency range of 0.5 to 45 Hz was employed to eliminate artifacts. Thirdly, the independent component analysis (ICA) method was performed to cancel irrelevant noise. Finally, the automatic artifact reject technique, artifact subspace reconstruction (ASR), in the EEGLAB toolbox (Delorme and Makeig, 2004), was used to exclude segments of data exceeding the conservative 0.5-s window standard deviation threshold of 17, considered as the maximum acceptable limit.

### Feature extraction

In this study, the EEG signals were first extracted to 4-s epochs with a 50% overlap, forming the foundational dataset population, which was subsequently employed for classification with being labeled as AD or CN. Then, three types of signal features of resting-state EEG, i.e., spectrum, complexity, and synchronization, were extracted for each epoch.

### Spectrum metrics

For time-domain metrics, the mean, variance, and interquartile range (IQR) were chosen as the features (Miltiadous et al., 2021). For a data segment $x_{j}$ with length N , the mean metric $\bar{x}$ , estimating the central tendency of a probability distribution for a variable, can be defined by:

$$\bar{x}=\frac{1}{N}\overset{N}{\underset{j=1}{\sum }}x_{j}$$

The variance metric Var , representing the width of data around its central value, can be defined by:

$$Var=\frac{1}{N-1}\overset{N}{\underset{j=1}{\sum }}{\left(x_{j}-\bar{x}\right)}^{2}$$

The IQR, the difference between $Q_{1}$ and $Q_{3}$ , referred to 25th percentile (lower) and 75th percentile (upper), respectively, can be calculated by:

$$IQR=Q_{3}-Q_{1}$$

For the frequency-domain metrics, firstly, the power spectral density (PSD) method was used for each 4-s epoch. Next, the PSD for the whole frequency range of 0.5–45 Hz can be also calculated. Then, the five basic EEG rhythms (namely delta of 0.5–4 Hz, theta of 4–8 Hz, alpha of 8–13 Hz, beta of 13–25 Hz, and gamma of 25–45 Hz) were obtained. Finally, to normalized processing, the relative band power (RBP) of each EEG rhythm was obtained by Miltiadous et al., (2023):


$$RBP_{i}=\;\frac{{\mathrm{Energy}}_{i}}{\sum {\mathrm{Energy}}_{i}},i=\delta ,\theta ,\alpha ,\beta ,\gamma$$

### Complexity metrics

Entropy measures typically quantify the degree of complexity and predictability of a signal (Coifman and Wickerhauser, 1992). In this study, the approximate entropy (ApEn), permutation entropy (PermEn), multiscale entropy (MSE), and sample entropy (SamplEn) were used to describe the complexity of the entire frequency spectrum.

ApEn is a non-linear method that can be utilized for quantifying the irregularity of a time series, which can be defined by:


$$\mathrm{ApEn}\left(m,r,N\right)=-\left[\varphi^{m+1}\left(r\right)-\varphi^{m}\left(r\right)\right]$$

where $\varphi^{m}\left(r\right)=\overset{N-m+1}{\underset{k=1}{\sum }}\frac{\ln c_{r}^{m}\left(k\right)}{N-m+1}$ , and $c_{r}^{m}\left(k\right)=\;\frac{\mathrm{count}\;\left[d\left(k,l\right)\leq r\right]}{N-m+1}$ is a correlation integer estimated by the distance $d\left(k,l\right)$ between the vectors $u\left(k\right)=\left[x\left(k\right),\;x\left(k+1\right),\ldots ,\;x\left(k+m-1\right)\right]$ and $u\left(l\right)$ . In this study, the pattern length m=1 and the similarity factor r=0.2 times the standard deviation of the time series (Burioka et al., 2005; Abásolo et al., 2009).

PermEn is a complexity measure of ordinal patterns for arbitrary, noisy, and large signals, which can be defined by:


$$\mathrm{PermEn}=-\int p\left(\pi \right)\log p\left(\pi \right)$$

where π represents all the permutations of order n , which corresponds to the number of embedding dimensions. $p\left(\pi \right)$ represents the probability associated with ordinal patterns π , indicating the relative frequency of ordinal patterns π (Bandt and Pompe, 2002). In this study, n was set as 3 (Tzimourta et al., 2019).

SamplEn is similar to ApEn but it excludes the assessment of self-similar patterns, which can be described by:


$$\mathrm{SamplEn}\left(m,r,N\right)=-\ln\frac{\varphi^{m}\left(r\right)}{\varphi^{m+1}\left(r\right)}$$

where $\varphi^{m}\left(r\right)=\overset{N-m+1}{\underset{i=1}{\sum }}\frac{\ln c_{r}^{m}\left(i\right)}{N-m+1}$ , and $c_{r}^{m}\left(i\right)=\;\frac{\mathrm{count}\;{\left[d\left(i,j\right)\leq r\right]}_{i\neq j}}{N-m+1}$ estimated the distance $d\left(k,l\right)$ between the vectors $u\left(k\right)=\left[x\left(k\right),\;x\left(k+1\right),\ldots ,\;x\left(k+m-1\right)\right]$ and $u\left(l\right)$ . Among them, m=2 and r=0.15 (Yang et al., 2013).

As a modification of SamplEn for the scaled signal, MSE introduces a range for multiple time scales denoted as τ , employed to create a coarse-grained version of the original time series, and each element of the coarse-grained signal can be calculated by:


$$y_{j}^{\left(\tau \right)}=\frac{1}{\tau }\overset{j\tau }{\underset{k=\left(j-1\right)\tau +1}{\sum }}x_{k},1\leq j\leq N/\tau$$

In our experiments, m=2,r=0.15, and τ=5 , which was consistent with previous studies (Costa et al., 2005; Yang et al., 2013).

### Synchronization metrics

Based largely on graph theory, recent developments in the analysis of signal synchronization have been rapidly developed (Liu et al., 2017). In this study, the four metrics of clustering coefficient, characteristic path length, efficiency, and small-worldness were used to describe the signal synchronization from complex brain network features (Bullmore and Sporns, 2009).

The clustering coefficient measures the number of connections among the immediate neighbors of a node, expressed as a proportion of the maximum number of possible connections (Demuru et al., 2020). The clustering coefficient $C_{i}$ of node i can be defined by:


$$C_{i}=\frac{2e_{i}}{k_{i}\cdot \left(k_{i}-1\right)}$$

where $e_{i}$ represents the number of edges in the neighborhood of node i , and $k_{i}$ representing the degree of node i is a basic feature of the number of connections that node i makes to other nodes.

The characteristic path length L is the minimum number of edges required to traverse from one node to another, which can be defined by Gaal et al. (2010):


$$L=\frac{1}{N\cdot \left(N-1\right)}\underset{i,j\in V,i\neq j}{\sum }l_{ij}$$

where N represents the number of all nodes, and $l_{ij}$ represents the minimum path length between notes i and j .Efficiency $E_{\mathrm{global}}$ exhibits an inverse relationship with path length, yet it is more straightforward to employ for estimating topological distances between elements of disconnected graphs, which can be defined by Buchel et al. (2021):


$$E_{\mathrm{global}}=\frac{1}{N\cdot \left(N-1\right)}\underset{i,j\in V,i\neq j}{\sum }\frac{1}{l_{i}}$$

The ‘small-world’ property is characterized by a combination of elevated local clustering among nodes within a network and abbreviated paths that establish global connections across the network. Small-worldness σ is thus determined by the ratio of the clustering coefficient to the path length (Liu et al., 2017):


$$\sigma =\frac{\gamma }{\delta }$$

where γ represents the standardized clustering coefficients, defined by the ratio of the clustering coefficient to the random network’s clustering coefficient, and δ represents the standardized characteristic path length, established as the ratio of characteristic path length to the random network’s characteristic path length.

### Classification algorithm

According to previous studies (Fiscon et al., 2018; Miltiadous et al., 2021; Safi and Safi, 2021), the supervised learning classification methods of decision trees, random forests, and SVM were used as the classifiers. For each algorithm, the leave-one-person-out cross-validation was used as the testing method (Miltiadous et al., 2021), where all epochs from a specific subject are designated as the test set, while the remaining epochs collectively form the training set. Then, the indexes of accuracy, sensitivity, and specificity were calculated, respectively, according to the following equations (Baratloo et al., 2015):


$$\begin{array}{c} \;\mathrm{Accuracy}=\frac{TP+TN}{TP+TN+FP+FN}\\ \;\mathrm{Sensitivity}=\frac{TP}{TP+FN}\\ \;\mathrm{Specificity}=\frac{TN}{TN+FP} \end{array}$$

where the variables TP, FP, TN, and FN represent true positive, false positive, true negative, and false negative, respectively.

## Results

### Signal characteristics

**Figure 1:**
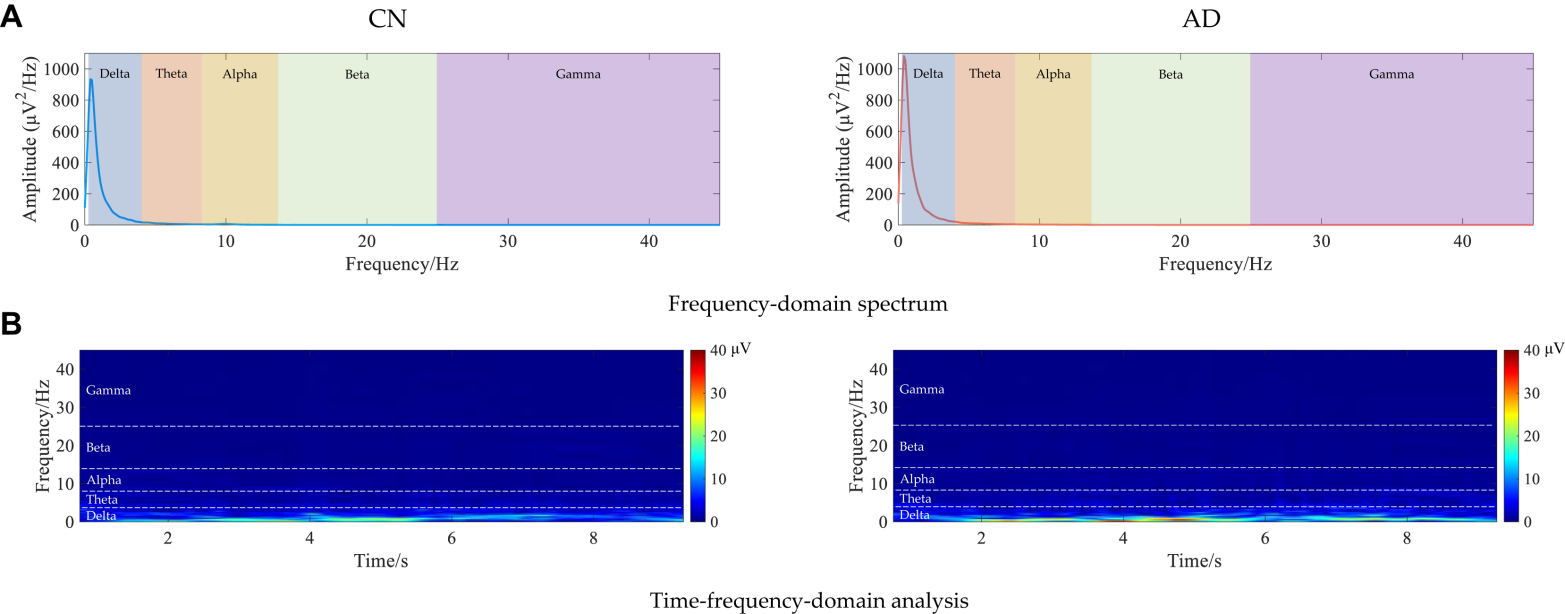
Examples of the frequency-domain and time-frequency-domain analyses of resting-state EEG for CN and AD subjects. **(A)** Frequency-domain spectrum. **(B)** Time-frequency-domain analysis.

To further analyze the spectrum characteristics of the signal, Figure 1 shows examples of the frequency-domain and time-frequency-domain analyses of resting-state EEG for CN and AD subjects. As shown in the frequency-domain spectrum and time-frequency-domain analysis of Figures 1A,B, there was some difference in the frequency spectrum EEG signals between CN and AD subjects, e.g., an increase in the delta rhythms in AD subjects.

**Figure 2:**
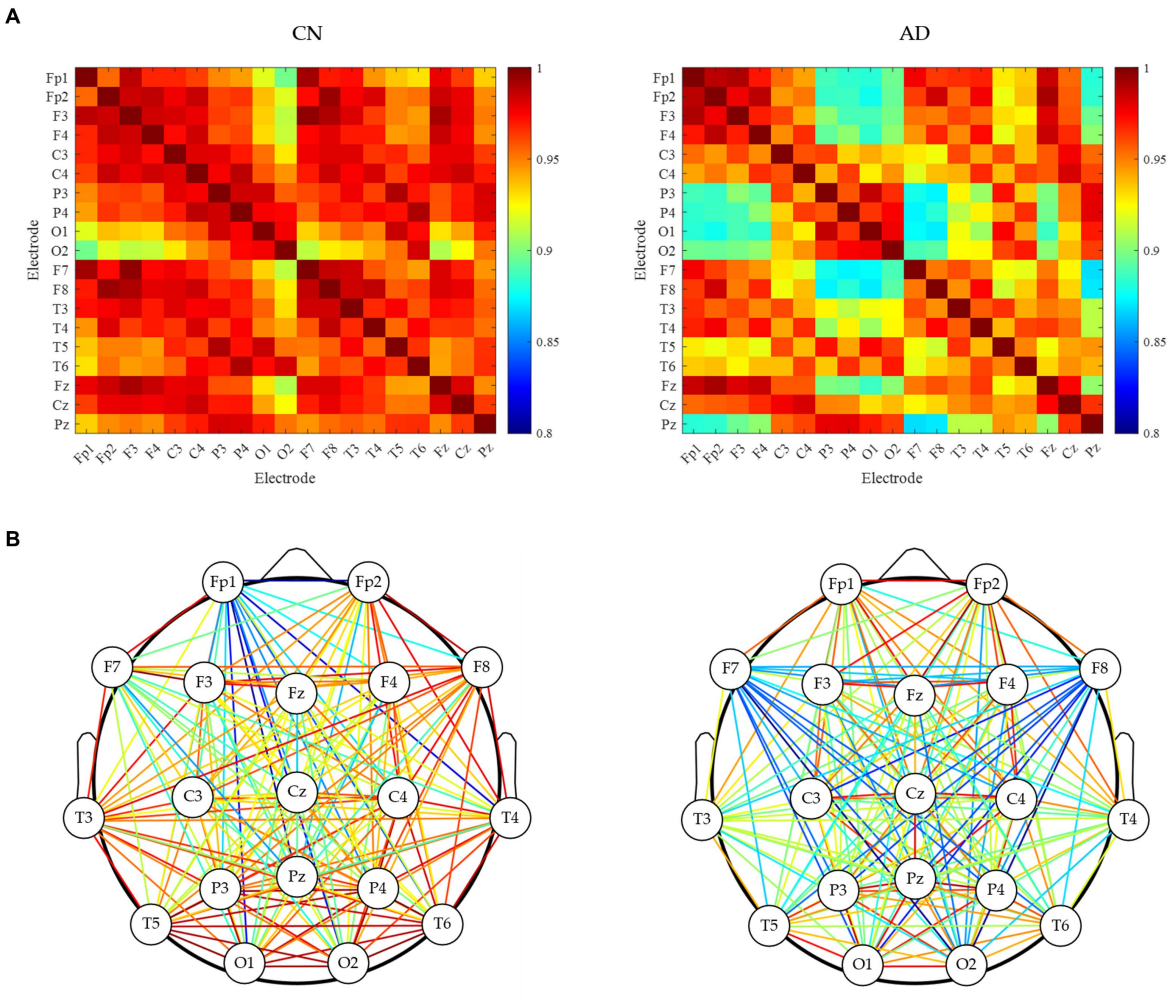
Brain network analysis of resting-state EEG for CN and AD subjects. **(A)** Correlation matrix between each electrode. **(B)** brain network connectivity.

Subsequently, the brain network analysis of resting-state EEG for CN and AD subjects was analyzed. As shown in Figure 2A, the correlation matrix between all pairs of electrodes was generated, indicating a decreasing correlation in AD subjects compared to CN subjects. As shown in Figure 2B, the analysis of the brain network gave clearer connectivity between all pairs of electrodes, showing that there was a decrease in brain network connectivity in AD subjects compared to CN subjects, indicating the decreased EEG synchrony in AD patients under rest conditions.

### Signal features

**Figure 3:**
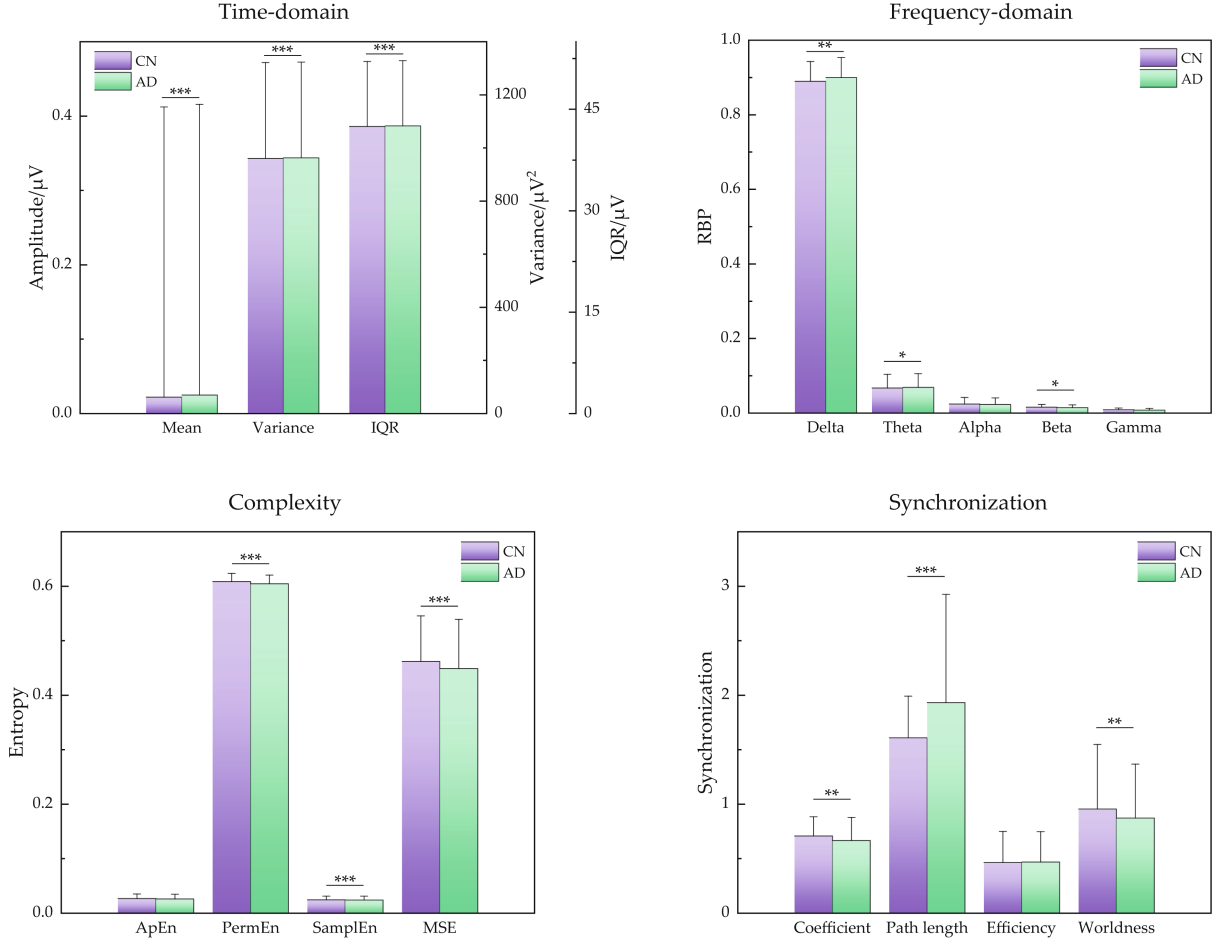
Signal features of time-domain, frequency-domain, signal complexity, and signal synchronization for CN and AD individuals. Statistics were assessed by independent samples t-test. ***p < 0.001; **p < 0.01; *p < 0.05.

For more statistical analysis of EEG signals between CN and AD subjects, the EEG data was first extracted to 4 s epochs with 50% overlap after being preprocessed for each subject, generating 14,515 epochs labeled AD from 36 AD subjects and 12,011 epochs labeled CN from 29 CN subjects. According to the difference between signal characteristics described above, the signal features of time-domain, frequency-domain, complexity, and synchronization were obtained for each epoch. Moreover, the mean and SD of these signal features are shown in Figure 3, and subsequently, their difference between AD and CN individuals was assessed by independent samples t-test.

For time-domain metrics, the mean, variance, and IQR demonstrated a little upward trend for AD subjects (*p* < 0.001, respectively). For frequency-domain metrics, the low-frequency bands of delta and theta showed a slight increase (*p* < 0.05, respectively), the high-frequency band of beta showed a slight decrease (*p* < 0.05), and the high-frequency bands of alpha and gamma showed a decreasing but insignificant trend, indicating that the major changes in the diagnosis of AD were the attenuated power in higher frequency bands (alpha, beta, and gamma) and increased power in lower bands (delta and theta), that is AD caused EEG signals to slow down. For complexity metrics, the entropies of PermEn, SamplEn, and MSE presented a low value in AD subjects (*p* < 0.001, respectively), revealing that EEG signals of AD showed reduced complexity and seemed to be regular. For synchronization metrics, the features of clustering coefficient and small-worldness demonstrated a decreasing tendency (*p* < 0.01, respectively) and characteristic path length demonstrated an increasing tendency (*p* < 0.001), showing decreased EEG synchrony in AD patients.

### Classification results

Using these EEG signal features, three classification algorithms of decision trees, random forests, and SVM were carried out to identify the AD and CN groups by the leave-one-person-out cross-validation. Table 1 presents the accuracy, sensitivity, and specificity results of three classification algorithms, showing that the random forest achieved the highest classification performance with an accuracy of 95.86%, and SVM performed the lowest accuracy of 88.54%.

**Table 1:** Accuracy, sensitivity, and specificity results of three classification algorithms with leave-one-person-out cross-validation.

	Accuracy	Sensitivity	Specificity
Decision tree	95.65%	95.91%	95.35%
Random forest	95.86%	96.41%	97.40%
SVM	88.54%	94.72%	81.23%

## Discussion

The presented study underscores the potential of integrating signal features from spectrum, complexity, and synchronization domains of resting-state EEG for enhancing the diagnosis of AD. This study achieved a higher classification accuracy performance of 95.86% for AD and CN subjects based on resting-state EEG, compared to previous studies using the same dataset with a classification accuracy of 77.01% (Miltiadous et al., 2023), showing the combination of these three types of EEG signal features can enhance the classification performance. Besides, in contrast to other studies, e.g., the classification accuracy of 78.50% (Miltiadous et al., 2021) and 83.30% (Fiscon et al., 2018), our study also showed a better performance.

By capturing diverse aspects of neural dysfunction, this integration of spectrum, complexity, and synchronization signal features may offer a more holistic understanding of the underlying pathology. Several key factors have been studied and explored in the pathological causes of AD, e.g., plaques composed of amyloid β, and tangles composed of hyperphosphorylated tau (Scheltens et al., 2021). According to the signal features shown in Figure 3, first, the power spectrum shifted from higher frequency components (alpha, beta, and gamma) toward lower frequency components (delta and theta), which may be related to loss of cholinergic innervations in AD patients (Cassani et al., 2018). Second, a decrease in the complexity of the brain’s electrical activity has been noted in AD patients. This phenomenon is potentially attributed to extensive neuronal loss and diminished connectivity in cortical regions, resulting in simpler EEG dynamics (Czigler et al., 2008). Third, reduced synchrony was also presented in AD patients, which can potentially be attributed to a functional disconnection within the neocortex, e.g., anatomical disconnections among different cortical regions in combination (Dauwels et al., 2010).

As for the validation method, this study adopted the leave-one-person-out cross-validation method. In contrast to k-fold cross-validation, which employs samples from the same participant in both training and test sets, the leave-one-person-out cross-validation method offers a more realistic validation strategy since no same-subject epochs were in both the training and the test set at the same time (Häfner et al., 2012; Isler et al., 2015).

Some limitations should also be paid attention in this study. First of all, this study only focused on the classification of AD and CN subjects. However, the severity of AD may affect EEG performance, and the severity, e.g., mild, moderate, and serious (Cassani et al., 2018), may also be classified in future studies. Next, the signal processing and feature extraction methods can also be further expanded. For example, the synchronization metrics may also be obtained by Granger causality (Babiloni et al., 2016), phase coherence (McBride et al., 2013), and state space synchrony (Wang et al., 2016), except for the mentioned methods in this study. Then, the features were obtained by averaging EEG signals across the whole recorded electrodes. Nevertheless, the cause of AD may arise from specific brain regions with variable effects on each channel’s EEG signals, and the average approach may not be very appropriate. Some techniques, e.g., EEG topographic map (Zheng et al., 2020), physiological cognition (Ranchet et al., 2017), and partial brain networks (Schöll, 2022), may be further carried out in future studies.

Based on prior research, researchers have computed an array of statistical characteristics from EEG recordings, e.g., cohesion (Lindau et al., 2003), wavelet analysis (Fiscon et al., 2018), and Hjorth parameters (Safi and Safi, 2021), which were subsequently employed to train their classification models. Moreover, in some studies, the basic EEG rhythms were further divided (Nishida et al., 2011). For example, the rhythm alpha was found as α1 (8–10 Hz) and α2 (10–12 Hz), and the rhythm beta was divided into β1 (12.5–18 Hz), β2 (18.5–21 Hz), and β3 (21.5–30 Hz) (Caso et al., 2012). Hence, in future studies, further division of EEG rhythms may be used in the frequency-domain metrics and entropies.

Another point the authors would like to mention was that the regional distribution of the brain of these features corresponding to AD was not always consistent for each EEG rhythm and each subject (Knyazeva et al., 2010; Tzimourta et al., 2019). Hence, future studies may focus on the detailed distribution of EEG to find the EEG source localization for AD pathogenesis, and then combine EEG signaling manifestations with causes of AD formation to achieve early detection of AD (Aghajani et al., 2013). Furthermore, the deep learning methods based on large databases can also be explored in future work to realize end-to-end prediction (Khojaste-Sarakhsi et al., 2022).

## Conclusion

The proposed integrated approach of three types of EEG signal features demonstrated promising results in differentiating AD patients from healthy controls. The fusion of spectrum, complexity, and synchronization features exhibited improved diagnostic accuracy compared to using individual features alone. This suggests that the combination of multi-domain features of EEG signals provides a more comprehensive representation of the neurophysiological changes associated with AD. This study recommended the integration of EEG features of spectrum, complexity, and synchronization for aiding the diagnosis of AD.

## Data availability statement

The original contributions presented in the study are included in the article/supplementary material, further inquiries can be directed to the corresponding authors.

## Ethics statement

The studies involving humans were approved by Scientific and Ethics Committee of AHEPA University Hospital, Aristotle University of Thessaloniki. The studies were conducted in accordance with the local legislation and institutional requirements. The participants provided their written informed consent to participate in this study.

## Author contributions

XZ: Formal analysis, Methodology, Software, Writing – original draft, Writing – review & editing. BW: Investigation, Writing – original draft. HL: Data curation, Writing – review & editing. WW: Validation, Writing – original draft. JS: Methodology, Project administration, Writing – original draft. WF: Project administration, Writing – original draft. RJ: Funding acquisition, Writing – original draft. YH: Formal analysis, Writing – review & editing. CJ: Conceptualization, Writing – review & editing. XW: Funding–acquisition, Project administration, Visualization, Writing – review & editing. SC: Conceptualization, Data curation, Resources, Writing – original draft.
